# Protein phosphatase 2A dysfunction in Alzheimer’s disease

**DOI:** 10.3389/fnmol.2014.00016

**Published:** 2014-03-11

**Authors:** Jean-Marie Sontag, Estelle Sontag

**Affiliations:** Faculty of Health and Medicine, School of Biomedical Sciences and Pharmacy, The University of NewcastleCallaghan, NSW, Australia

**Keywords:** Alzheimer’s disease, amyloid, LCMT1, methylation, phosphorylation, protein phosphatase 2A, tau

## Abstract

Protein phosphatase 2A (PP2A) is a large family of enzymes that account for the majority of brain Ser/Thr phosphatase activity. While PP2A enzymes collectively modulate most cellular processes, sophisticated regulatory mechanisms are ultimately responsible for ensuring isoform-specific substrate specificity. Of particular interest to the Alzheimer’s disease (AD) field, alterations in PP2A regulators and PP2A catalytic activity, subunit expression, methylation and/or phosphorylation, have been reported in AD-affected brain regions. “PP2A” dysfunction has been linked to tau hyperphosphorylation, amyloidogenesis and synaptic deficits that are pathological hallmarks of this neurodegenerative disorder. Deregulation of PP2A enzymes also affects the activity of many Ser/Thr protein kinases implicated in AD. This review will more specifically discuss the role of the PP2A/Bα holoenzyme and PP2A methylation in AD pathogenesis. The PP2A/Bα isoform binds to tau and is the primary tau phosphatase. Its deregulation correlates with increased tau phosphorylation *in vivo* and in AD. Disruption of PP2A/Bα-tau protein interactions likely contribute to tau deregulation in AD. Significantly, alterations in one-carbon metabolism that impair PP2A methylation are associated with increased risk for sporadic AD, and enhanced AD-like pathology in animal models. Experimental studies have linked deregulation of PP2A methylation with down-regulation of PP2A/Bα, enhanced phosphorylation of tau and amyloid precursor protein, tau mislocalization, microtubule destabilization and neuritic defects. While it remains unclear what are the primary events that underlie “PP2A” dysfunction in AD, deregulation of PP2A enzymes definitely affects key players in the pathogenic process. As such, there is growing interest in developing PP2A-centric therapies for AD, but this may be a daunting task without a better understanding of the regulation and function of specific PP2A enzymes.

## THE PUZZLING DIVERSITY OF THE “PP2A” FAMILY

Protein phosphatase 2A (PP2A) refers to a ubiquitous, highly conserved family of at least 96 serine/threonine phosphatases that represent 0.1-1% of total cellular proteins and play a crucial role in regulating most cellular functions (Reviewed in Virshup and Shenolikar, 2009). The typical and primary mammalian holoenzyme is a heterotrimeric complex between the catalytic C subunit (PPP2CA or PPP2CB isoforms), a scaffolding A subunit (PPP2R1A or PPP2R1B isoforms) and a regulatory B subunit, which belongs to one of four distinct families: B (PPP2R2), B’ (PPP2R5), B” (PPP2R3), or B”’/striatins (PPP2R4). B subunits are encoded by 15 different genes, and 23 isoforms have been identified so far. A comparatively much smaller amount of endogenous dimeric [AC] core enzymes, and PP2A complexes containing the catalytic C subunit coupled to the alpha four subunit or other PP2A-specific modulators, have also been isolated *in vivo* (Reviewed in Sents et al., 2013).

Although “PP2A” represents a major portion of Ser/Thr phosphatase activity in most tissues, genetic studies have pointed to the important observation that each PP2A isoform likely exerts non-redundant cellular functions (Zhao et al., 1997). Consequently, scientists should ideally refrain from using ambiguous and imprecise statements, such as “PP2A regulates this” or “activation of PP2A does that,” as those do not take into account the exquisite diversity and functional selectivity of PP2A enzymes. Indeed, despite the tremendous functional significance of the “PP2A” family, isoform-specific substrates remain in large part to be characterized *in vivo*, including in the central nervous system (CNS). The unfortunate lack of valid isoform-specific PP2A antibodies, inhibitors/activators and activity assays, the complexity of PP2A regulation, and the fact that most “PP2A” enzymes play essential cellular functions have all historically combined to hinder progress in dissecting the role of each specific member of the “PP2A” family.

## THE MIND-BLOWING SOPHISTICATION OF “PP2A” REGULATION

**FIGURE 1:**
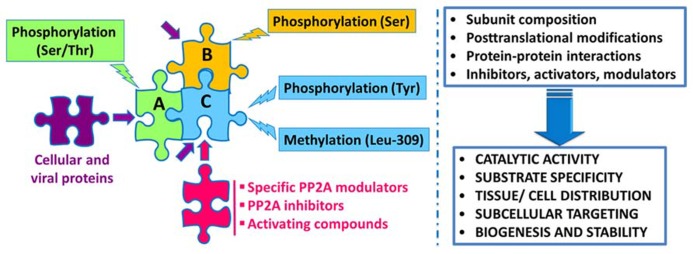
**Schematic overview of the intricate regulation of PP2A enzymes.** Major PP2A holoenzymes of this very large family (>96 enzymes) are heterotrimers containing a scaffolding “A” (one of two isoforms), a catalytic “C” (one of two isoforms), and one variable regulatory “B” subunit (one of twenty three isoforms). PP2A subunits are subjected to post-translational modifications, including methylation of the catalytic subunit on a conserved Leucine-309 residue, and phosphorylation. Endogenous subunit interactions, interaction of PP2A subunits with a variety of viral and cellular proteins, and binding of specific PP2A inhibitors and modulatory proteins to the catalytic subunit, all combine to modulate PP2A catalytic activity and ensure PP2A isoform-specific targeting and substrate specificity. Specific modulatory proteins also critically regulate PP2A biogenesis and stability. In addition, many compounds are known to enhance PP2A catalytic activity. See text for details.

Protein phosphatase 2A regulation is highly complex, involving not only the interplay of specific regulatory subunits and modulators, but also post-translational modifications, protein–protein interactions and subcellular compartmentalization (**Figure 1**). Notably, these interwoven regulatory processes coalesce to ensure PP2A isoform-specific functional specificity, as described below:

### PP2A POST-TRANSLATIONAL MODIFICATIONS

Protein phosphatase 2A catalytic subunit is uniquely methylated on Leu-309 by the dedicated leucine carboxyl methyltransferase-1 (LCMT-1; Lee et al., 1996; De Baere et al., 1999). Like all methyltransferases, LCMT1 activity depends on the supply of the universal methyl donor, *S*-adenosylmethionine (SAM), and is inhibited by *S*-adenosylhomocysteine (SAH; Leulliot et al., 2004; Sontag et al., 2007). Conversely, the PP2A-specific methylesterase PME-1 can directly bind to the active site of the catalytic subunit, remove the methyl group and inactivate PP2A by evicting manganese ions required for phosphatase activity (Xing et al., 2008). Protein complexes containing PME-1 coupled to demethylated, inactive PP2A have been isolated *in vivo* (Ogris et al., 1999). Methylation is thought to play a critical role in the biogenesis of PP2A holoenzymes. Many groups have reported that altering the overall methylation status of PP2A catalytic subunit can lead to changes in cellular PP2A subunit composition, and in turn, substrate specificity (Reviewed in Janssens et al., 2008). Significantly, down-regulation of LCMT1 expression leads to a significant decrease of PP2A methylation and concomitant loss of PP2A holoenzymes containing the regulatory Bα (or PPP2R2A) subunit (PP2A/Bα; Lee and Pallas, 2007; Sontag et al., 2008; MacKay et al., 2013). PP2A enzymes can also become transiently inactivated following tyrosine phosphorylation of the catalytic subunit at the putative Tyr-307 site, via activation of src kinase, epidermal growth factor receptor or insulin signaling (Chen et al., 1992). Other modifications of PP2A C subunit include ubiquitination, which targets PP2A for degradation (McConnell et al., 2010), and tyrosine nitration that increases PP2A activity in endothelial cells (Wu and Wilson, 2009). Adding another layer of complexity to the regulation of PP2A holoenzymes, protein kinase A-mediated serine phosphorylation of selective PPP2R5A and PPP2R5D regulatory subunits belonging to the B’ family can also modulate PP2A catalytic activity (Ahn et al., 2007; Kirchhefer et al., 2014). A recent report also indicates the existence of regulated phosphorylation of the scaffolding A subunit on Ser/Thr residues, which affects its binding to the catalytic subunit and PP2A signaling in the heart (Kotlo et al., 2014). Sequence analyzes predict additional post-translational modifications in PP2A subunits that remain to be validated and characterized.

### DIRECT CONTROL OF PP2A CATALYTIC ACTIVITY

Natural toxins such as okadaic acid, calyculin, and fostriecin (Reviewed in Swingle et al., 2007), and endogenous nuclear inhibitors called I_1_^PP2A^ and I_2_^PP2A^/SET (Li and Damuni, 1998), can directly bind to the catalytic subunit and inhibit the phosphatase activity of the entire family of PP2A enzymes. Conversely, many endogenous small molecules, comprising metal cations, ceramides and polyamines, can enhance the activity of PP2A enzymes (Reviewed in Voronkov et al., 2011).

### REGULATORY PP2A SUBUNITS AND PP2A MODULATORS

Subunit interactions within the PP2A enzymatic complex can directly modulate PP2A catalytic activity (Kamibayashi et al., 1991). Regulatory B subunits play a distinctively important role in controlling PP2A substrate specificity. For instance, the Bα subunit specifically and markedly facilitates dephosphorylation of tau by PP2A (Sontag et al., 1996; Xu et al., 2008). Furthermore, direct interaction of PP2A catalytic subunit with specific regulatory proteins, including PME-1, LCMT1, the alpha4 subunit, and the PP2A phosphatase activator PTPA, critically modulates PP2A biogenesis and stability. Together, those also participate in the complex surveillance mechanisms that prevent untimely, detrimental PP2A activation within cells (Reviewed in Sents et al., 2013).

### PROTEIN–PROTEIN INTERACTIONS

Through one or more of their composing subunits, PP2A enzymes interact with a wide variety of cellular proteins (For example see Sontag, 2001), including protein kinases, receptors, cytoskeletal proteins and transcription factors, as well as viral proteins (Reviewed in Guergnon et al., 2011). Of particular relevance to the Alzheimer’s disease (AD) field, PP2A/Bα holoenzymes can directly bind to the microtubule-associated protein tau (Sontag et al., 1999, 2012; Xu et al., 2008). PP2A enzymes can also associate with protein kinases that have been linked to AD, such as glycogen synthase kinase 3β (GSK3β) and cyclin-dependent kinase 5 (cdk5; Plattner et al., 2006), and neuronal receptors, e.g., the NMDA receptor (Chan and Sucher, 2001) and the metabotropic glutamate receptor 5 (Mao et al., 2005; Arif et al., 2014).

### SUBCELLULAR COMPARTMENTALIZATION

Protein phosphatase 2A regulatory subunits are expressed in a cell- and tissue-specific manner, which is responsible for the isoform-specific distribution of PP2A in the brain (Strack et al., 1998). They can also influence the subcellular localization of PP2A either directly or indirectly by binding to specific intracellular proteins. For instance, some B’ subunits target PP2A to the nucleus (McCright et al., 1996) or the centrosome (Flegg et al., 2010); Bα subunits can direct some PP2A pools to microtubules, which could serve as a cytoskeletal reservoir of inactive enzymes (Sontag et al., 1995; Hiraga and Tamura, 2000). PP2A regulatory proteins can further affect the phosphatase distribution. For example, PME-1 stabilizes a nuclear pool of inactive PP2A enzymes (Longin et al., 2008), while methylation by LCMT1 influences the amounts of PP2A enzymes bound to plasma membrane microdomains (Sontag et al., 2013). Yet, much remains to be learned about the precise mechanisms that control PP2A translocation to discrete subcellular compartments. In any case, the regulated formation of specific PP2A isoform-containing multi-protein scaffolds likely contributes to the general spatiotemporal control of many signaling pathways in the CNS.

## THE MULTIFACETED DEREGULATION OF “PP2A” IN AD

**FIGURE 2:**
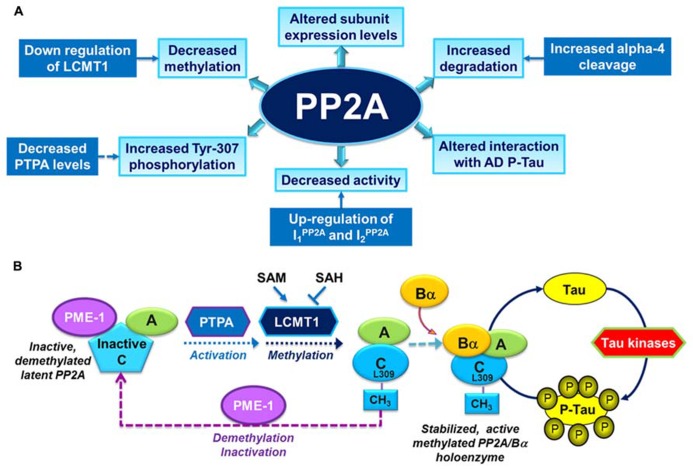
**Overview of PP2A dysfunction in AD and its link with the deregulation of tau.** **(A)** Altered PP2A subunit expression, activity and post-translational modifications have been described in AD autopsy brain tissue. Some of these changes may be mediated by alterations in specific PP2A modulatory proteins (LCMT1, PTPA, alpha4) and endogenous PP2A inhibitors (I_1_^PP2A^ and I_2_^PP2A^) that have also been reported in AD autopsy brain tissue. They also decrease the interaction of PP2A with tau. **(B)** The biogenesis of the PP2A/Bα holoenzyme, the primary Ser/Thr tau phosphatase *in vivo*, is believed to be controlled by Leu-309 methylation of PP2A catalytic subunit by LCMT1. This reaction requires the supply of SAM, the universal methyl donor, and is inhibited by SAH. The PP2A methylesterase, PME-1, can demethylate and inactivate PP2A through distinct mechanisms, and form a complex with inactive PP2A enzymes. Those inactive complexes could be-reactivated via the action of the PP2A activator PTPA, allowing for subsequent methylation of PP2A C subunit. Many brain Ser/Thr protein kinases, including GSK3β, oppose the action of PP2A/Bα and promote tau phosphorylation. Inhibition and/or down-regulation of PP2A can enhance tau phosphorylation directly by preventing its dephosphorylation, or indirectly by up-regulating tau kinases.

Over the past years, several alterations in PP2A and PP2A regulatory enzymes have been identified in AD autopsy brain tissue (**Figure 2A**), and are described as follows:

### ALTERATIONS IN PP2A

There is a significant decrease in total PP2A activity measured in AD cortical and hippocampal brain homogenates (Gong et al., 1993; Gong et al., 1995; Sontag et al., 2004b). Deficits in PP2A activity are in line with the reported down-regulation of PP2A catalytic C subunit at the gene (Loring et al., 2001), mRNA (Vogelsberg-Ragaglia et al., 2001) and protein (Sontag et al., 2004b) expression levels in AD. In contrast, “PP2A” expression levels are increased in AD astrocytes (Pei et al., 1997). More specifically, decreased expression levels of PP2A regulatory Bγ (or PPP2R2C) and B’ε (or PPP2R5E) subunit mRNAs in the hippocampus (Vogelsberg-Ragaglia et al., 2001), and cortical Bα subunit (Sontag et al., 2004b) have been reported in AD. Notably, the loss of neuronal PP2A/Bα holoenzymes correlates with the down-regulation of PP2A methylation and severity of phosphorylated tau (P-tau) pathology in AD-affected brain regions (Sontag et al., 2004a, b). Lastly, increased phosphorylation of PP2A at Tyr-307 has been found in P-tau-rich, tangle-bearing neurons from post-mortem brain (Liu et al., 2008b).

### ALTERATIONS IN PP2A REGULATORY PROTEINS

Significantly, down-regulation of LCMT1 protein expression parallels the deficits in PP2A methylation observed in AD (Sontag et al., 2004a). Up-regulation of I_1_^PP2A^ and I_2_^PP2A^, and mislocalization and cleavage of I_2_^PP2A^, could underlie the inactivation of PP2A in AD neocortical neurons (Tanimukai et al., 2005). Decreased expression levels of PTPA in AD brain tissue may also lead to inactivation of PP2A by indirectly increasing levels of PP2A phosphorylated at the Tyr-307 site (Luo et al., 2013). Lastly, increased calpain-mediated cleavage of alpha4, which critically modulates PP2A stability, could be responsible for increased degradation of PP2A catalytic subunit in AD (Watkins et al., 2012).

Collectively, those studies point to a central role for PP2A dysfunction in AD pathogenesis. However, the primary mechanism(s) responsible for such multilayered and confounding deregulation of PP2A enzymes and modulators remain obscure.

## *IN VIVO* IMPAIRMENT OF “PP2A” LEADS TO AD-LIKE PATHOLOGY AND COGNITIVE DEFECTS

*In vivo* use of phosphatase inhibitors such as okadaic acid has been shown in many studies to induce cognitive impairment and widespread neurotoxic effects that are reminiscent of the hallmark pathological processes occurring in AD pathology, i.e., the accumulation of P-tau, amyloidogenesis, synapse loss and neurodegeneration (Malchiodi-Albedi et al., 1997; Arendt et al., 1998; Sun et al., 2003; Kamat et al., 2013). However, these inhibitors lack specificity toward PP2A at the concentrations used *in vivo* (Swingle et al., 2007). Their usefulness is also dramatically limited by their inherent lack of selectivity toward specific PP2A isoforms. Not surprisingly, inhibition of total cellular PP2A activity ultimately leads to neuronal cell death.

More specific and targeted manipulation of PP2A through experimental overexpression and knock-down of particular subunits and/or modulatory proteins, has more convincingly demonstrated the implication of PP2A in AD. Knock-down of PP2A catalytic subunit (Kins et al., 2001) or PP2A B’δ (or PPP2R5D) regulatory subunit (Louis et al., 2011), and expression of the methylation-site L309A C subunit mutant (Schild et al., 2006) all induce AD-like tau phosphorylation in transgenic mice. Genetic studies in Drosophila also confirm the critical role played by PP2A in modulating P-tau toxicity (Steinhilb et al., 2007). Moreover, expression of an I_2_^PP2A^ fragment can recapitulate AD-like pathology in rat brain (Wang et al., 2010). However, similarly to exogenous inhibitors, I_2_^PP2A^ indiscriminately inhibits all PP2A isoforms, including enzymes that may be irrelevant to the neurodegenerative process in AD.

## THE INTIMATE INTERRELATIONSHIP BETWEEN DEREGULATION OF “PP2A” AND AD BRAIN PROTEIN KINASES

It is well recognized now that the imbalance between protein kinases and phosphatases is a major contributor to the neurodegenerative process in AD. At pretty much each crossroad of major signal transduction pathways, there are dedicated PP2A enzymes ready to ambush and dephosphorylate selective Ser/Thr kinase(s). Yet again, for each protein kinase/protein phosphatase couple, the precise nature of the PP2A isoform involved is not always known. Specific PP2A inhibition has been proven to lead to *in vivo* deregulation of many major brain Ser/Thr kinases implicated in AD, including GSK3β (Wang et al., 2010; Louis et al., 2011), cdk5 (Louis et al., 2011; Kimura et al., 2013), extracellular signal-regulated kinase (ERK) and JNK (Kins et al., 2003). In fact, PP2A inhibition can override the inhibition of these key AD-tau kinases (Planel et al., 2001). Apart from affecting tau phosphorylation, abnormal activation of GSK3β, cdk5, and ERK has been linked to cytoskeletal abnormalities (microtubules, neurofilaments), alterations in amyloid precursor protein (APP) phosphorylation and processing, impairment of neurogenesis, alterations in synaptic plasticity and induction of apoptotic processes (Reviewed in Crews and Masliah, 2010; Medina and Avila, 2013, 2014). Thus, by deregulating the activity of central AD protein kinases, PP2A dysfunction can promote aberrant stimulation of signaling cascades that contribute to neuronal and synaptic damage in AD.

Conversely, some Ser/Thr protein kinases can in turn modulate PP2A. For instance, activated GSK3β has been reported to induce PP2A inactivation via several mechanisms: phosphorylation of PP2A on Tyr307 (Yao et al., 2011); demethylation of PP2A on Leu309 through inhibition of LCMT1 and up-regulation of PME1 (Yao et al., 2012); and accumulation of I_2_^PP2A^ (Liu et al., 2008a). Besides Ser/Thr kinases, the protein tyrosine kinase src promotes the phosphorylation of PP2A on Tyr-307, resulting in PP2A inactivation and subsequent tau phosphorylation (Xiong et al., 2013; Arif et al., 2014). Nonetheless, the interconnection between the deregulation of these various protein kinase/ PP2A-dependent signaling cascades and AD pathogenesis is still unclear.

## DEREGULATION OF PP2A/Bα: A MAJOR CONTRIBUTOR TO PHOSPHO-TAU PATHOLOGY

Notably, “PP2A” mediates ~71% of total tau phosphatase activity in the human brain (Liu et al., 2005). While many PP2A holoenzymes have the potential to indirectly affect tau phosphorylation by modulating key tau protein kinases (For example see Louis et al., 2011), biochemical and structural studies have demonstrated that PP2A/Bα is the primary PP2A isoform that mediates tau dephosphorylation (Sontag et al., 1996, 1999; Xu et al., 2008). Specific inhibition of PP2A/Bα is associated with enhanced tau phosphorylation at many AD-like phosphoepitopes, and subsequent inability of tau to bind to and stabilize microtubules (Sontag et al., 1996). Deregulation of PP2A/Bα alone also affects microtubule stability (Nunbhakdi-Craig et al., 2007) and neurite outgrowth (Sontag et al., 2010) in neuroblastoma cells. Since PP2A/Bα is a major brain enzyme, its loss has also the potential to deregulate many neuronal signaling pathways. As described earlier, it is especially significant that the biogenesis of PP2A/Bα holoenzymes is intimately related to the methylation state of PP2A. Accordingly, the phosphorylation state of tau is vulnerable to neuronal changes in PP2A methylation/demethylation processes (**Figure 2B**).

**FIGURE 3:**
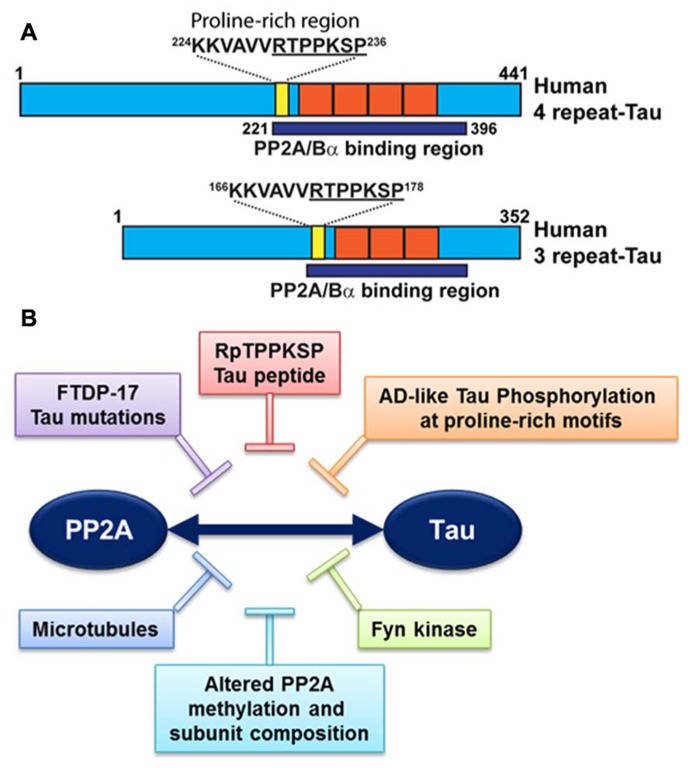
**Deregulation of PP2A-tau protein–protein interactions in AD. (A)** The PP2A/Bα holoenzyme can directly interact with three- or four-repeat human tau isoforms via a domain encompassing the microtubule-binding repeats (orange), resulting in tau dephosphorylation. A specific proline-rich motif (yellow) that contains the Thr231 phosphorylation site plays a critical role in modulating PP2A-tau protein–protein interactions. **(B)** PP2A-tau protein–protein interaction can be inhibited *in vitro *by: (1) Alterations in tau, including AD-like phosphorylation and FTDP-17 missense mutations; (2) decreased expression levels of PP2A methylation and PP2A/Bα in AD; (3) Fyn kinase and pseudophosphorylated RpTPPKSP peptides. Disruption of normal PP2A-tau interactions is predicted to affect tau phosphorylation state and function.

It is noteworthy that PP2A/Bα can directly bind to tau via a domain encompassing the microtubule-binding of tau; this interaction maximizes the efficiency of tau dephosphorylation by PP2A (Sontag et al., 1999; Xu et al., 2008; **Figure 3A**). PP2A/Bα has more affinity for adult four-repeat tau than three-repeat tau isoforms (Sontag et al., 1999). Inactive pools of PP2A/Bα are also directly associated with microtubules, so that binding to tau and dephosphorylation of tau could in principle only occur when both tau and PP2A fall off the microtubule cytoskeleton (Sontag et al., 1999; Hiraga and Tamura, 2000). Notably, tau missense mutations found in frontotemporal dementia with Parkinsonism linked to chromosome 17 (FTDP-17; Goedert et al., 2000) and AD-mimicking tau phosphorylation in proline-rich motifs (Eidenmuller et al., 2001) inhibit the association of tau with PP2A (**Figure 3B**). Recently, we identified a specific RTPPKSP Proline-rich motif in tau that critically modulates PP2A-tau protein–protein interactions (Sontag et al., 2012). Phosphorylation of the Thr-231 residue in this motif markedly decreases the affinity of tau for PP2A. This is potentially physiologically significant since phosphorylation of tau at Thr-231, a target site for ERK2, GSK3β, and cdk5, occurs early in AD and can further inhibit the ability of PP2A/Bα to dephosphorylate other major AD-tau phosphoepitopes (Landrieu et al., 2011). Moreover, the RTPPKSP motif is also a binding site for SH3 domain-containing proteins including Fyn, a protein tyrosine kinase that phosphorylates tau on Tyr residues and plays a major role in AD pathogenesis (Lee, 2005). Indeed, Fyn kinase and PP2A compete for tau binding *in vitro *(Sontag et al., 2012). Conversely, decreased PP2A methylation and PP2A/Bα levels in AD will disrupt normal PP2A-tau interactions (Sontag et al., 2007), thereby preventing PP2A-mediated tau dephosphorylation while allowing for enhanced binding of Fyn kinase or other regulators to the tau proteins. In theory, this could lead to a net increase in tau phosphorylated at both Ser/Thr and Tyr sites, as observed in AD; in turn, abnormally elevated levels of phosphorylation will disrupt tau localization and function (Reviewed in Martin et al., 2011). These *in vitro* studies underscore the critical importance of tau as a scaffold for key signaling molecules implicated in AD. Indeed, the significance of disrupting these tau protein–protein interactions for deregulation of tau and neuronal homeostasis has been largely underestimated to date.

## THE EMERGING SIGNIFICANCE OF ALTERED PP2A METHYLATION IN AD

**FIGURE 4:**
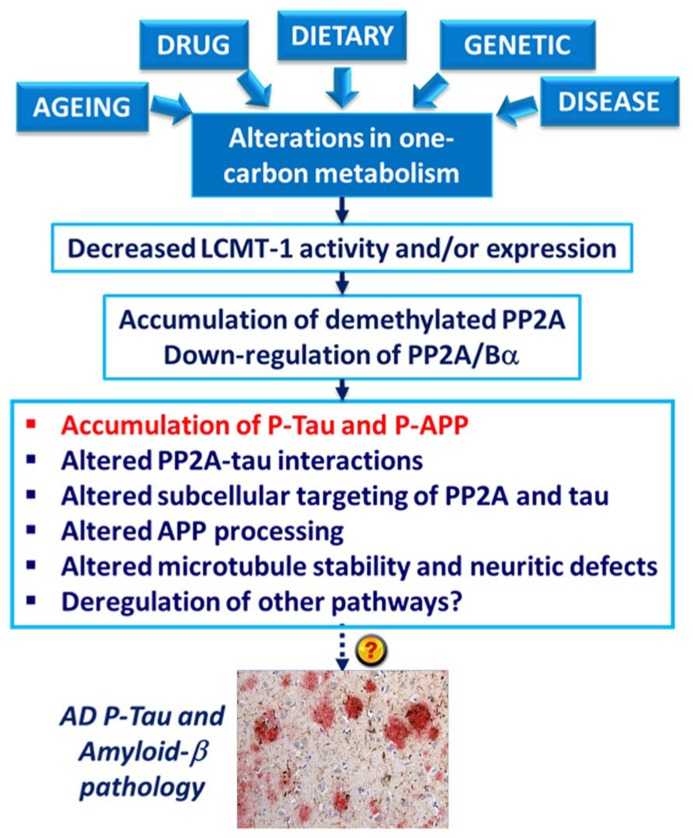
**Model for the link between alterations in one-carbon metabolism, deregulation of PP2A methylation and AD-like pathology.** Dietary B-vitamin deficiency, genetic polymorphisms in key enzymes that metabolize folate and homocysteine, drugs (e.g., the anti-folate drug, methotrexate), diseases (e.g., liver disease) and aging can all lead to impairment of one-carbon metabolism. In turn, alterations in the methylation cycle result in decreased LCMT1 activity and/or expression levels, and subsequent down-regulation of PP2A methylation and PP2A/Bα holoenzymes. This is associated with the accumulation of phosphorylated tau and APP proteins *in vivo*. Experiments in cultured cells and *in vitro* also link alterations in neuronal PP2A methylation with disruption of PP2A-tau protein–protein interactions, alterations in the subcellular targeting of PP2A and tau, APP processing, microtubule stability, and neuritic defects.

The deregulation of PP2A methylation in AD is especially interesting, not only because it can lead to a loss of PP2A/Bα, a major tau regulator, but also because PP2A methylation state is intimately linked to the integrity of one-carbon metabolism, which regulates SAM supply (Reviewed in Fowler, 2005). It is well documented that hyperhomocysteinemia, and low folate/ B12-vitamin status that independently lead to elevations of homocysteine levels, can induce disturbances in the brain methylation potential. Such alterations are associated with increased risk for sporadic AD and cognitive decline in humans (Reviewed in Selhub et al., 2010; Zhuo et al., 2011; Bottiglieri, 2013). Remarkably, impairment of one-carbon metabolism in animal models can reproduce AD-like pathological features: accumulation of P-tau (Sontag et al., 2007; Zhang et al., 2008; Wei et al., 2011); enhanced amyloidogenesis (Pacheco-Quinto et al., 2006; Zhang et al., 2009; Zhuo et al., 2010; Zhuo and Pratico, 2010); increased phosphorylation of APP at the regulatory Thr-668 site (Sontag et al., 2007; Zhang et al., 2009); increased sensitivity to amyloid toxicity (Kruman et al., 2002); and cognitive impairment (Bernardo et al., 2007; Wei et al., 2011; Rhodehouse et al., 2013). Many of these effects appear now to be mediated in part by deregulation of PP2A (**Figure 4**). Dietary folate and B-vitamin deficiency (Sontag et al., 2008; Nicolia et al., 2010) and elevated homocysteine levels (Sontag et al., 2007, 2013; Zhang et al., 2008) lead to down-regulation of PP2A methylation and concomitant phosphorylation of tau and/or APP *in vivo*. In cultured cells, deregulation of PP2A methylation also affects APP processing (Sontag et al., 2007), neurite outgrowth (Sontag et al., 2010) and tau distribution (Sontag et al., 2013). It is worth mentioning that besides PP2A, other pathways can contribute to the link between impaired one-carbon metabolism and AD-like neurodegeneration. For instance, altered methylation of genes responsible for production of amyloid-β peptides is associated with enhanced amyloidogenesis (Fuso and Scarpa, 2011). In this context, it is especially interesting that age-related epigenetic changes in genes that participate in methylation homeostasis have been identified in late-onset AD (Wang et al., 2008).

## CONCLUDING REMARKS AND PERSPECTIVES: THE TAXING CHALLENGE OF DEVELOPING PP2A-CENTRIC THERAPEUTIC APPROACHES FOR AD

Altogether, there is overwhelming evidence from experimental studies that “PP2A” dysfunction is a key player in the development of P-tau pathology in AD. Few experimental studies also point to a link between “PP2A” deregulation and amyloidogenesis, but underlying mechanisms remain sketchy. PP2A enzymes also oppose the activity of many brain protein kinases up-regulated in AD. Consequently, there is growing interest in developing PP2A-targeted therapies for AD, especially to counteract P-tau pathology. Many compounds are already known to modulate PP2A activity by several direct and indirect mechanisms (Voronkov et al., 2011). *In vivo *studies have shown that some experimental compounds and drugs currently used clinically can “activate PP2A” and subsequently reverse AD-like tau phosphorylation, for instance: sodium selenate, by increasing “PP2A activity” through unknown mechanisms (van Eersel et al., 2010); the anti-AD drug, memantine, by targeting I_2_^PP2A^ (Chohan et al., 2006); and the anti-diabetic drug, metformin, by interfering with PP2A degradation (Kickstein et al., 2010). Thus, can simply “PP2A activators” be the next cure for AD and other tauopathies (Voronkov et al., 2011)?

We argue that there are still many hurdles to overcome to develop valid PP2A-based therapies. First, it is clear that the deregulation of PP2A and PP2A regulatory proteins in AD is multidimensional and intrinsically complex (**Figure 2A**), begging the question as to what is the primary mechanism of PP2A dysfunction that should be preferentially targeted in clinical trials? It is unlikely that a single compound will overcome all the many-sided PP2A deficits that are present in AD. It is not clear either how exactly pharmacological “PP2A (re)activation” will restore the collective function of specific PP2A holoenzymes and PP2A modulators that become down-regulated at the protein level in AD neurons. Next, as reported for clinical AD kinase inhibitors, specificity and side-effects are likely to be a huge issue, due to the broad spectrum of PP2A enzymes, their profuse abundance and their essential role. While PP2A/Bα is the primary tau phosphatase, none of the compounds tested so far have demonstrated isoform specificity, so that they are likely to interfere with the regulation of a plethora of PP2A enzymes that are irrelevant to the overall neurodegenerative process. Drugs may also target PP2A enzymes outside the brain and in brain regions not primarily involved in the disease process. The regulation of PP2A is still not well understood, and effects of indiscriminate and long-term “PP2A” activation or interference with vital PP2A-regulatory mechanisms are not known. Furthermore, pathological brain changes manifest at least one decade before appearance of AD symptoms (Sperling et al., 2011). Thus, one needs to take into account that treatment with pharmaceutical PP2A drugs may require to be started early and prolonged for a long period of time, which increases chances for compensatory mechanisms and side-effects. Lastly, the observation that PP2A-targeting compounds can improve AD-like pathology derives from testing in mouse models that do not reproduce the complexity of the pathology encountered in their human counterparts. For instance, the mice utilized do not exhibit the multifaceted aspects of PP2A deregulation that are inherent to AD autopsy tissue. More significantly, they do not exhibit the metabolic deficits observed in AD patients. For instance, epidemiological studies show that AD patients have low plasma folate status (Lopes da Silva et al., 2013), which can negatively impact PP2A methylation (Sontag et al., 2008). Therapeutic approaches may also need to take into account the existence of common human polymorphisms (Naj et al., 2010; Hua et al., 2011; Coppede et al., 2012; Mansouri et al., 2013) and age-related epigenetic changes (Wang et al., 2008) in folate-related genes that affect folate status and have been associated with late-onset AD. The resulting impairment of methylation pathways may offset the efficacy of potential PP2A agonists. In this context, it is worth mentioning that dietary compounds that target one-carbon metabolism and therefore influence PP2A methylation, such as betaine (Chai et al., 2013), SAM (Fuso et al., 2012), and B-vitamin supplementation (Zhang et al., 2008; Wei et al., 2011), all improve AD-like pathological features *in vivo*. While promising, the validity of these therapeutic approaches for AD needs to be further investigated in clinical trials (Morris, 2012; Bottiglieri, 2013). In conclusion, it is paramount to gain a better understanding of the regulation and function of specific PP2A isoforms in normal neuronal homeostasis to guarantee success of novel PP2A-centric therapeutic strategies for AD.

## AUTHOR CONTRIBUTIONS

Jean-Marie Sontag and Estelle Sontag contributed equally to this work.

## Conflict of Interest Statement

The authors declare that the research was conducted in the absence of any commercial or financial relationships that could be construed as a potential conflict of interest.
